# Characterization of Abscisic Acid and Ethylene in Regulating the White Blush in Fresh-Cut Carrots

**DOI:** 10.3390/ijms232112788

**Published:** 2022-10-24

**Authors:** Chichang Chen, Ye Wang, Li Xu, Siwen Wang, Xiangning Chen, Yuanyue Shen

**Affiliations:** 1 Beijing Key Laboratory of Agricultural Product Detection and Control for Spoilage Organisms and Pesticides, Food Science and Engineering College, Beijing University of Agriculture, Beijing 102206, China; 2 Beijing Key Laboratory for Agricultural Application and New Technique, College of Plant Science and Technology, Beijing University of Agriculture, Beijing 102206, China; 3 Key Laboratory of Agricultural Product Processing and Quality Control (Co-Construction by Ministry and Province), Ministry of Agriculture and Rural Affairs, Beijing 102206, China; 4 Research Institute of Processing Preservation and Circulation of Agricultural Products, Beijing 102206, China

**Keywords:** white blush, carotenoids, lignin, molecular mechanisms

## Abstract

The surface of fresh-cut carrots is apt to white blush, however the physiological and molecular mechanism for this process is not yet fully understood. In this study, exogenous abscisic acid (ABA) and ethylene separately promoted and inhibited the white-blush formation after three days after treatment, respectively. Metabolome analysis found that white-blush components mainly consist of p-hydroxyphenyl lignin and guaiacyl lignin. Transcriptome analysis found an increase in the whiteness values was consistent with the higher expression of genes encoding O-methyltransferase, trans-anol O-methyltransferase, bergaptol O-methyltransferase, caffeic acid 3-O-methyltransferase, phenylalanine ammonia-lyase, and ferulate-5-hydroxylase, together with the lower expression of genes encoding cinnamic acid 4-hydroxylase caffeoyl-CoA O-methyltransferase and 5-O-(4-coumaroyl)-D-quinate 3′-monooxygenase. In conclusion, ABA plays an important role in lignin biosynthesis essential to the formation of white blush in fresh-cut carrots. This is the first report that uncovers the physiological and molecular causes of white blush in fresh-cut carrots, providing a basis for white-blush control in fresh-cut carrots.

## 1. Introduction

Carrot (*Daucas carota*) is known as a yellow or clove radish with a unique flavor, rich nutrition, and beneficial compounds for health [1]. Moreover, fresh-cut carrots occupy an important role in the fresh-cut market. However, the surface of fresh-cut carrots is apt to white blush during storage, finally resulting in a short shelf life [2]. Moreover, the physiological and molecular mechanisms underlying the white blush of fresh-cut carrots remain not fully understood.

In the past years, several studies preliminarily explored the control of whitening n fresh-cut carrots [3]. Chen et al. [3] reported that H_2_S treatment decreased the contents of H_2_O_2_ and malondialdehyde (MDA), as well as the activities of lignin biosynthesis related enzymes, including phenylalanine ammonia lyase (PAL), polyphenol oxidase (PPO), and peroxidase (POD). Ranjitha [2] found that the shelf-life of fresh-cut carrots can be extended to 12 days by coating carrot slices with polyvinyl alcohol or pectin mainly through preventing the formation of white blush and the changes in color, texture, and flavor during storage at 8 °C. Our previous studies found that 0.6% citric acid and 0.4% ascorbic acid could reduce the whiteness value of fresh-cut carrots [4]. These findings, however, discuss exogenous components involved in physiologic reactions, extended quality retention, and process optimization, and how they might restrict the whiteness of fresh-cut carrots. Regarding the cause of whiteness, early reports suggest that cut carrots are apt to lost water, making the surface color light and white. Moreover, the cutting causes the rupture of most cells in the cut surface, resulting in phenylpropionic acid metabolism, lignin synthesis, and whiteness on the damaged surface [5]. However, the defined molecular mechanism remains unknown.

It was previously reported that lignin biosynthesis is first involved in the formation of hydroxycinnamoyl-CoA esters, as a common precursor of monolignols, which is synthesized by cinnamoyl-CoA reductase, pointing to the conversion of coumaroyl-, feruloyl-, and sinapoyl-CoAs to coumaraldehyde, coniferaldehyde, and sinapaldehyde, respectively [6]. In general, lignin as a complex polymer is composed of p-hydroxyphenyl (H), guiacyl (G), and sinapyl (S) units that are derived from the phenylpropanoids-pathway intermediates, which polymerize to form a secondary cell wall structure essential to biotic and abiotic stresses [7]. Notably, a report finds that the enzymes key to lignin monomer synthesis, such as C4H, 4CL, CCoAOMT, and CAD, show cell-specific expression patterns [8]. Especially, it was found that lignin served as a barrier to maintain cell osmotic balance and membrane integrity, reducing water loss [9]. Notably, it was recently found that abscisic acid (ABA) regulates secondary cell-wall formation and lignin deposition in *Arabidopsis thaliana* [10]. All data available suggest that the white blush in fresh-cut carrots might be related to ABA.

In addition, in the past years, it was determined that fruit color transition in the model plants tomato and strawberry is trigged by ethylene (Eth) and abscisic acid, respectively [11,12]. Additionally, the significance of ABA as a terpenoid phytohormone was identified as playing a key role in controlling plant development and its bioactive compounds with hypoglycemic potential [13,14]. In the present study, we explored the composition of fresh-cut carrot whitening substances and the effect of abscisic acid and ethylene on whitening and carotenoids/lignin contents. We uncover the roles of ABA and Eth on the whitening, which results from the phenylpropionic acid metabolism involved in lignin biosynthesis. This is the first report on the physiological and molecular mechanism for the white blush of fresh-cut carrots, providing new insights into the processes.

## 2. Results

### 2.1. Effects of ABA and Eth on Whiteness Index of Fresh-Cut Carrot

**Figure 1:**
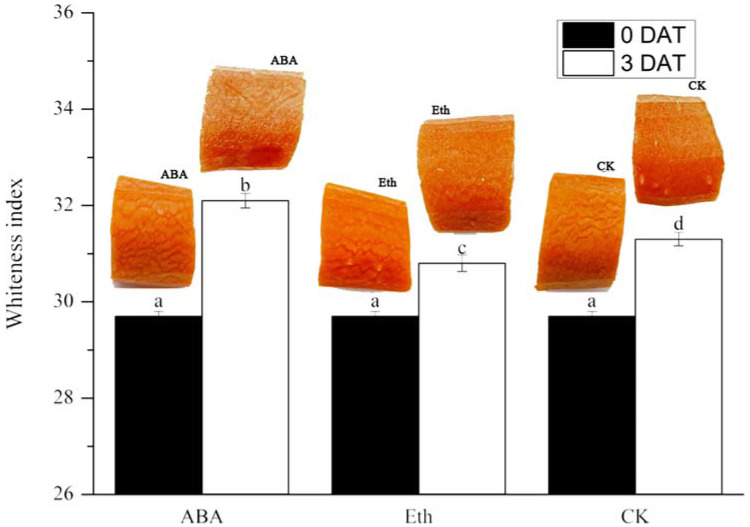
Whiteness index of each treatment group in fresh-cut carrot. DAT: days after treatment; ABA: the fresh-cut carrot was treated with abscisic acid; Eth: the fresh-cut carrot was treated with ethylene; and CK: the fresh-cut carrot was treated with cleaning water. Each value is presented as the mean ± SE (n = 3). Means with different letters are significantly (*p* < 0.05) different between each sample type for different treatment groups.

To explore the effect of ABA and ethylene on whitening, the cut carrots were divided into three groups and soaked in 50 mg/L ABA, 500 mg/L ethephon, and water (CK group) for 5 min, respectively. As shown in Figure 1, with storage time extension, the whiteness appeared clear at three DAT (days after treatment), and as to the whiteness values, ABA group > CK group > Eth group. These results demonstrate that ABA and Eth promoted and inhibited the whitening in comparison to the CK, respectively, suggesting that ABA might play a role in the whiteness conformation.

### 2.2. Metabolome Analysis of Effects of ABA and Eth on Carotenoids in Fresh-Cut Carrot

**Figure 2:**
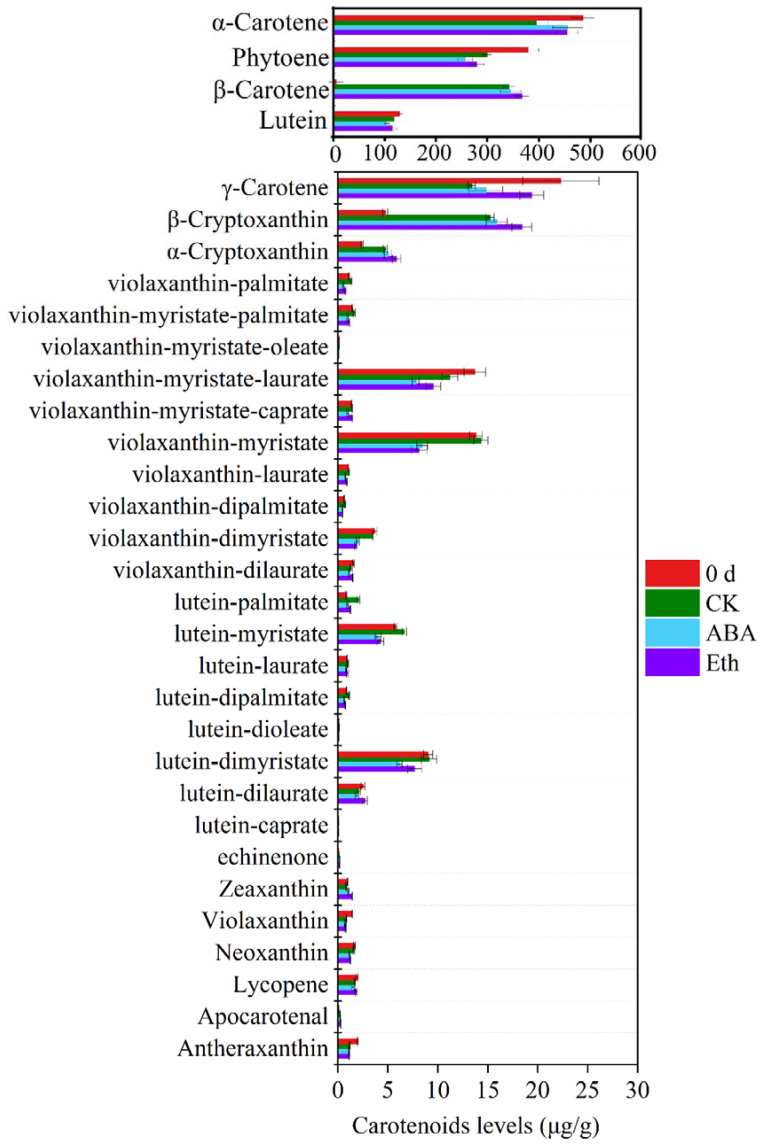
Metabolome analysis of effects of ABA and Eth on carotenoids.

**Figure 3:**
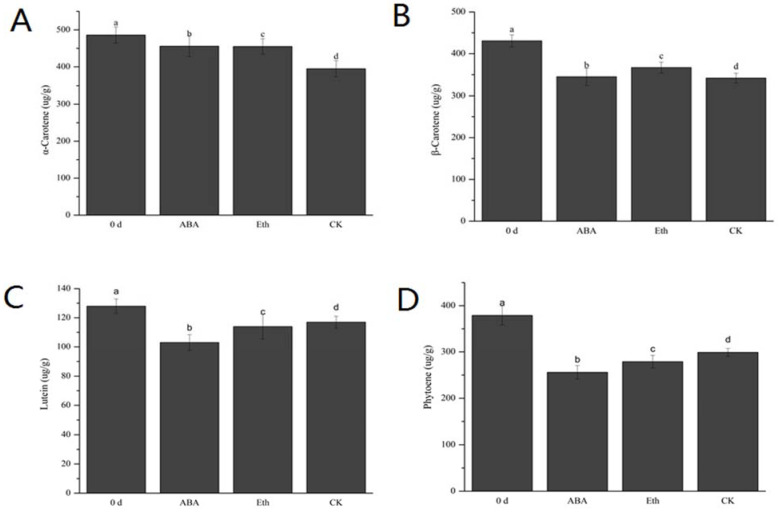
The content of carotenoids in each treatment group of fresh-cut carrot. Eth represents the ethylene treatment group, ABA represents the abscisic acid treatment group, and CK represents the water treatment group. The surface tissues were taken on the 3rd day of carrot extraction. (**A**) Effects of each treatment on α-carotene content, (**B**) Effects of each treatment on β-carotene content, (**C**) Effects of each treatment on lutein content, and (**D**) Effects of each treatment on phytoene content. Each value is presented as the mean ± SE (n = 3). Means with different letters are significantly (*p* < 0.05) different between each sample type for different treatment groups.

To explore molecule components of the whiteness, metabolome analysis was conducted using ABA-treated and ethylene-treated fresh-cut carrots and water treatment was used as the CK group. Carotenoids were yellow, orange–red, or red polyene compounds, generally composed of eight isoprenoid units [15]. In order to accurately determine the content of carotenoids in each treatment group, the standard curves of different substances were plotted, as shown in the Supplementary Materials, Table S1. The contents of carotenoids in each group were determined accurately, as shown in the Supplementary Materials, Table S2. The surface tissue of fresh-cut carrots was mainly made up of α-carotene, β-carotene, phytoene, and lutein (Figure 2 and Figure 3). Compared with 0 d, the contents of α-carotene, β-carotene, phytoene, and lutein in each group decreased after 3 DAT. During storage, the degree of whiteness of fresh-cut carrots increased, which indicated that the main substance causing the whiteness of fresh-cut carrots was not carotenoids. Therefore, the decline in carotenoids during storage may be related to the synthesis of ABA.

### 2.3. Metabolome Analysis of Effects of ABA and Eth on Lignins in Fresh-Cut Carrot

In order to compare lignin contents in different samples, the mass spectrum peaks of each metabolite in different samples were corrected according to the retention time and peak type of metabolites, so as to ensure the accuracy of quantitative analysis. Based on the metabolic database, sample metabolites were qualitatively and quantitatively analyzed by mass spectrometry.

Lignins were complex phenylpropane monomer polymers, which were synthesized through the phenylpropionic acid pathway, starting from the deamination of phenylalanine to form cinnamic acid [16]. After a series of hydroxylation, methylation, and reduction reactions, three major monomers were finally generated, including coumaryl alcohol, coniferyl alcohol, and sinapyl alcohol. According to the different monomers, lignin could be divided into three types: syringyl lignin (S-lignin) prepared by polymerization of syringyl propane monomers; guaiacyl lignin (G-lignin) synthesized by polymerization of guaiacyl propane monomers; and hydroxy-phenyl lignin (H-lignin) synthesized by polymerization of p-hydroxyphenyl propane monomers [17].

**Figure 4:**
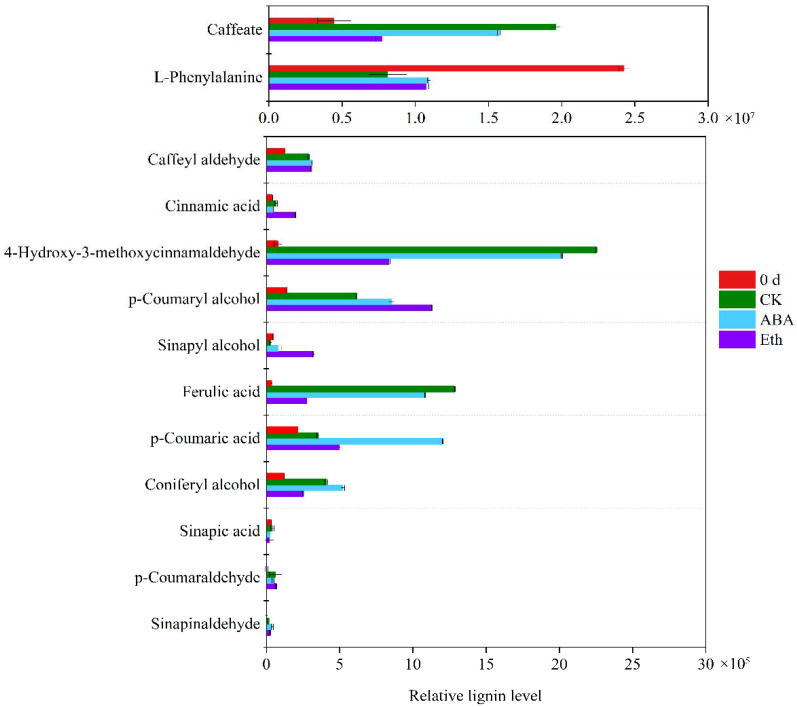
Metabolome analysis of effects of ABA and Eth in lignins contents.

Based on the metabolome that metabolites with fold change ≥ 2 were selected as the final difference metabolites (Figure 4). The substances detected in the lignin pathway and the difference of metabolites were shown in the Supplementary Materials, Tables S3 and S4. In contrast, the ABA and Eth treatment by 3 d resulted in an intense whiteness value when compared with the control (Figure 1). Compared with the 0 day group, the content of L-phenylalanine in the CK group decreased, and the contents of sinapinaldehyde, p-coumaraldehyde, coniferyl alcohol, caffeate, ferulic acid, p-coumaryl alcohol, 4-Hydroxy-3-methoxycinnamaldehyde, and caffeyl aldehyde all increased. Compared with the CK group, the contents of sinapyl alcohol, sinapinaldehyde, and p-coumaric acid increased in the ABA treatment group, while the contents of other substances did not change significantly. Compared with the CK group, the contents of sinapic acid, caffeate, ferulic acid, and 4-Hydroxy-3-methoxycinnamaldehyde decreased in the Eth treatment group, while the contents of sinapyl alcohol and cinnamic acid increased.

**Figure 5:**
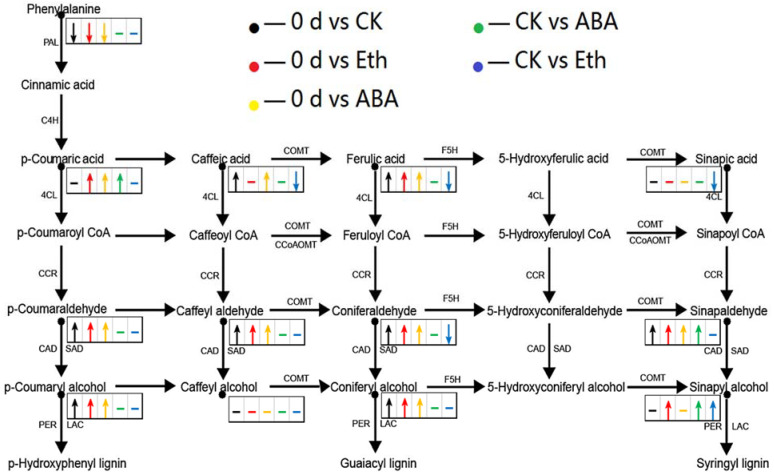
Contents and changes in lignins in fresh-cut carrot in various treatments. ↓: the metabolite content was significantly down; −: the metabolite content had no significant change; and ↑: the metabolite content was significantly up.

It can be observed in Figure 4 that the contents of sinapinaldehyde, p-coumaraldehyde, coniferyl alcohol, ferulic acid, p-coumaryl alcohol, 4-hydroxy-3-methoxycinnamaldehyde, and caffeyl aldehyde in ABA, CK, and Eth groups were higher than those in the 0 day group, showing an obvious change in lignin contents during storage. Given that p-coumaryl alcohol was synthesized by p-hydroxyphenyl lignin, while coniferyl alcohol was synthesized by guaiacyl lignin, the main lignin monomers in the surface substances of fresh-cut carrot were p-hydroxyphenyl lignin and guaiacyl lignin. These results demonstrate that the white blush in fresh-cut carrot is attributed to lignin accumulation as a result of cutting-derived stress. We investigated the differential accumulated metabolites between the different groups based on the fold change ≥ 2 or fold change ≤ 0.5. A total of 9, 10, 10, 3 and 5 differential accumulated metabolites were recorded at 0 d vs. CK, 0 d vs. Eth, 0 d vs. ABA, CK vs. ABA, and CK vs. Eth, respectively (Figure 5).

### 2.4. Transcriptome Analysis of Effects of ABA and Eth on Lignin-Related Gene Expression in Fresh-Cut Carrot

In order to understand the molecular mechanism of the white blush on fresh-cut carrots, RNA-seq was performed using various fresh-cut carrots by the expression differences of three biological replicates. Pearson’s correlation coefficient was used as the evaluation index of biological repeat correlation. The closer r was to 1, the stronger the correlation between the two duplicate samples and the statistical chart of correlation between each group of samples was drawn, as shown in the Supplementary Materials, Figure S1.

It can be seen from the Supplementary Materials, Figure S2, that compared with the 0 day group, the gene expression levels of the ABA group, CK group, and Eth group were significantly different. As shown in A~C, compared with the 0 day group, there were 2795 up-regulated genes and 3198 down-regulated genes in the ABA group, 3141 up-regulated genes and 3347 down-regulated genes in the CK group, and 2647 up-regulated genes and 3969 down-regulated genes in the Eth group. Based on the annotation of KEGG gene and enrichment analysis of KEGG gene, the pathway in the KEGG database was taken as the unit, and a hypergeometric test was applied to determine the pathway which was significantly enriched in the differential expression gene compared with the whole genome background. This screened out the pathway associated with the white blush of fresh-cut carrots.

**Figure 6:**
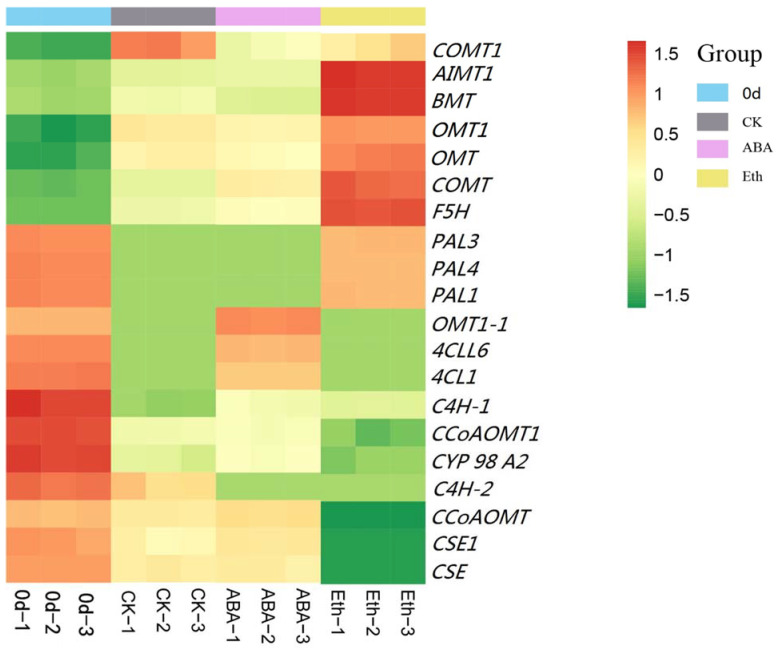
Clustering heat map of differentially expressed genes. COMT: caffeic acid 3-O-methyltransferase; AIMT1: trans-anol O-methyltransferase 1; BMT: Bergaptol O-methyltransferase; OMT: O-methyltransferase; F_5_H: ferulate-5-hydroxylase; PAL: pheylalanine ammonia-lyase; 4CLL6: 4-coumarate—CoA ligase-like 6; 4CL: trans-cinnamate 4-monooxygenase; C4H: cinnamic acid 4-hydroxylase; CCoAOMT: caffeoyl-CoA O-methyltransferase; CYP 98 A2: cytochrome P450 98A2; and CSE: caffeoylshikimate esterase.

As can be seen in Figure 6, compared with the 0 day group, the genes encoding *C4H-1*, *C4H-2*, *CCoAOMT*, *CCoAOMT1*, *CYP98A2*, *CSE1*, and *CSE* in the CK group were all down-regulated in transcripts, while the genes encoding *OMT1*, *OMT*, *AIMT1*, *BMT*, *COMT*, *COMT1*, and *F5H* were all up-regulated in transcripts; the genes encoding *C4H-1*, *4CLL6*, *4CL*, *CCoAOMT*, *CCoAOMT1*, *CYP98A2*, *CSE1,* and *CSE* in the ABA group were all down-regulated in transcripts, while the genes encoding *OMT1*, *OMT*, *AIMT1*, *BMT*, *OMT1-1*, *COMT*, *COMT1,* and *F5H* in the ABA group all were up-regulated in transcripts; and the genes encoding *C4H-1*, *CCoAOMT*, *CYP98A2*, *PAL3*, *PAL4*, and *PAL1* all down-regulated and the genes encoding *OMT1*, *OMT*, *AIMT1*, *BMT*, *COMT*, *COMT1,* and *F5H* all were up-regulated in transcripts in the Eth group. The expression of genes encoding *OMT1*, *OMT*, *AIMT1*, *BMT*, *COMT*, *COMT1,* and *F5H* in the CK group, ABA group, and the Eth group were all up-regulated; while the expression of genes encoding *C4H-1*, *CCoAOMT*, and *CYP98A2* in the three groups were all down-regulated. In combination with the changes of the whiteness value, it was shown that the increased transcripts of the gene encoding *OMT*, *AIMT*, *BMT*, *COMT*, *PAL,* and *F5H*, together with the decreased transcripts of the gene encoding *C4H*, *CCoAOMT*, and *CYP98A*, contribute to the white blush of fresh-cut carrots.

Compared with the CK group, the expression of genes encoding *C4H-1*, *CCoAOMT*, *CYP98A2*, *CSE*, *AIMT1*, *COMT,* and *F5H* were up-regulated and the genes encoding *OMT1*, *OMT*, *BMT*, and *COMT1* were all down-regulated in the ABA group; the expression of genes encoding *C4H-1*, *OMT1*, *OMT*, *AIMT1*, *BMT*, *COMT*, *COMT1*, and *F5H* were all up-regulated and the genes encoding *CCoAOMT*, *CYP98A2,* and *PAL4* were both down-regulated in the Eth group. In combination with the change in their whiteness values, it appeared that the decreased expression of genes encoding *CCoAOMT* and *CYP98A,* and *PAL4* together with the increased expression of gene encoding *OMT*, *BMT*, and *COMT* delayed the white blush of fresh-cut carrots. Compared with the Eth group, the expression of the gene encoding *PAL* was increased in the ABA group, indicating that which was essential to the white blush. Taken together, these results suggest that *PAL* was a key gene in the cause of the white change. Meanwhile, inhibition of *CCoAOMT* and *CYP98A2* expression and promotion of *OMT*, *BMT, F5H*, and *COMT* expression contribute to delay the occurrence of the white blush in fresh-cut carrots.

## 3. Discussion

Fresh-cut fruits and vegetables have become more and more important in the vegetable processing industry because of their advantages for convenient use, high quality and safety, nutrition, and health [18]. However, compared with whole fruits and vegetables, fresh-cut fruits and vegetables cause physiological and biochemical changes, tissue browning, nutrient loss, and excessive microorganisms due to tissue structure destruction [19,20]. In general, there are two types of browning: enzymatic browning and non-enzymatic browning. After cutting, in some foods enzymatic browning is simple to generate, such as potatoes, apples, pears, and lotus root. It is easy to produce enzymatic browning after cutting in these foods. Browning of fruits and vegetables has recently aroused considerable interest among researchers. It is generally considered that the polyphenols in fresh-cut fruits and vegetables are oxidized to form quinones, and then dehydrated and polymerized to form dark brown substances, which cause the browning of fruits and vegetables in coordination with PPO and other enzymes [20]. However, some fruits and vegetables contain fewer phenolic substances, resulting in less browning, however, other physiological and biochemical changes can still occur, such as the whitening of carrots. Fresh-cut carrots will produce whitening during storage. At present, there is little research on the white blush of fresh-cut carrots, and there is little research on the specific substance of the surface whitening of fresh-cut carrots. The cause of the white blush of fresh-cut carrots has been speculated, but it has not been confirmed. In the present study, we found that the white blush on the surface of fresh-cut carrots was lignin, not carotenoids. This is based on an investigation of the compound composition of the cutting surface of the 0 d and 3 d fresh cut carrots (at the time of the white blush occurrence), together with ABA and Eth treatment as well as omics.

After fresh-cut carrots were treated with water, when whitening occurred 3 DAT, the contents of sinapinaldehyde, p-coumaraldehyde, coniferyl alcohol, caffeate, ferulic acid, p-coumaryl alcohol, 4-Hydroxy-3-methoxycinnamaldehyde, and caffeoyl aldehyde were found to increase and L-phenylalanine was found to decrease. This indicated that the white blush on the surface of fresh-cut carrots was mainly p-hydroxyphenyl lignin and guaiacyl lignin (Figure 5). From the perspective of phenylpropanoid biosynthesis, by comparing the gene expression between the CK-group and 0 d-group, it was found that the expressions of the genes encoding *C4H-1*, *C4H-2*, *CCoAOMT*, *CCoAOMT1*, *CYP98A2*, *CSE1,* and *CSE* were negatively correlated, while the expressions of the genes encoding *OMT1*, *OMT*, *AIMT1*, *BMT*, *COMT*, *COMT1,* and *F5H* were positively correlated (Figure 6). Compared with the CK group, the treatment with ABA promoted the growth of syringyl lignin and guaiacyl lignin (Figure 5). The gene encoding *C4H-1, CCoAOMT, CYP98A2, CSE, AIMT, COMT,* and *F5H* all up-regulated and the gene encoding *OMT1, OMT, BMT, and COMT1* all down-regulated in transcripts (Figure 6). The treatment with Eth promoted the growth of syringyl lignin (Figure 5). The genes encoding *OMT1, OMT, AIMT1, BMT, COMT, COMT1,* and *F5H* all up-regulated and the genes encoding *CCoAOMT, CYP98A2,* and *PLA4* all down-regulated (Figure 6). Compared with the ABA group, the treatment with Eth promoted the growth of syringyl lignin and inhibited the growth of guaiacyl lignin (Figure 5). The genes encoding *OMT1, OMT, BMT, BMT1, COMT, COMT1,* and *F5H* all up-regulated and the genes encoding *CCoAOMT, CYP98A2, PAL3, PAL4*, and *PAL1* all down-regulated (Figure 6). It was reported that ABA could promote the process of lignification [21]. Some studies suggested that oxidase was involved in the process of corking [22] and lignification [23]. Ethylene could reduce the activity of enzymes related to reactive oxygen species’ metabolism. Ethylene might affect the production of lignin by reducing the activity of enzymes related to reactive oxygen species’ metabolism [24,25]. The specific reasons for this affect need to be further explored. Compared with each treatment group, the lowest whiteness values were found in Eth-treated and the highest whiteness values were found in ABA-treated fresh-cut carrots, indicating that the increased *PAL* expression leads to serious white change. Changes in whiteness values and gene expression were analyzed, indicating PAL was a key gene in the white change, meanwhile, inhibition of *CCoAOMT* and *CYP98A2* expression and promotion of *OMT, BMT*, *F5H*, and *COMT* expression contributed to the delay in the occurrence of the white blush in fresh-cut carrots. 

Recent research results showed that the total amount of lignin synthesis was closely related to the production and activity of PAL, C4H, and 4CL [26]. The specificity of lignin was closely related to F5H, COMT, and CCoAOMT, which played a key role in the proportion of monomer lignin structure [27]. The amount of PAL enzyme generated in the Eth group was the lowest in the present study, indicating that the lignin content of fresh-cut carrots determines the whiteness value. Sewalt et al. [28] found that in transgenic tobacco that inhibited the expression of *PAL* or *C4H*, not only did the content of lignin decrease, but the S/G ratio of components also changed. Humphreys et al. [27] and Osakabe et al. [29] suggested that *CCoAOMT* might be mainly involved in the synthesis of the guaiacyl-lignin precursor, and *COMT* was mainly involved in the synthesis of the syringyl-lignin precursor. Compared with the CK group and the ABA group, the genes encoding *CCoAOMT*, *CYP98A2,* and *PAL4* down-regulated and the genes encoding *COMT* and *F5H* all up-regulated. The content of the syringyl lignin increased and the content of the guaiacyl lignin decreased (Figure 5 and Figure 6), which was consistent with the report. Changes in whiteness values and the total amount of lignin and the specificity of lignin were analyzed, indicating the increase in the S/G ratio could reduce the degree of whitening of fresh-cut carrots. 

The white blush in fresh-cut carrots during storage was an emergency response of the damaged bodies to protect themselves. Although the white blush was conducive to prevent the invasion of diseases and pests, it had a very bad impact on its commercialization, which is a problem that needs to be solved for the effective storage of fresh-cut carrots. In this study, we found that the component of the white blush in fresh-cut carrots was lignin, and the ratio of syringyl lignin to guaiacyl lignin would affect the degree of white blush in fresh-cut carrots, which gave us ideas for controlling the white blush in fresh-cut carrots. We could reduce the whiteness value of fresh-cut carrots by regulating the key enzymes of lignin monomer biosynthesis and changing the content and ratio of syringyl lignin and guaiacyl lignin, so as to improve the commercial value of fresh-cut carrots. Phenylpropanoid biosynthesis is a complex process. The regulation of key enzymes in the phenylpropanoid biosynthesis pathway may control the content and composition of lignin, which not only contributes to understanding the regulation of phenylpropanoid biosynthesis, but also helps to improve fruit quality related to phenylpropanoid biosynthesis, such as stone cell formation in pear fruits.

## 4. Materials and Methods

### 4.1. Plant and Reagents

We used carrots from Beinong market in Beijing, China. Abscisic acid (chromatographic grade, ≥98%) and ethephon (chromatographic grade, ≥90%) were purchased from Shanghai Macklin Biochemical Co., Ltd. (Shanghai, China). Methanol, ethanol, and acetonitrile were purchased from Merk Ltd. (Darmstadt, Germany). BHT (chromatographic grade, ≥99%) was purchased from Shanghai Aladdin Biochemical Technology Co., Ltd. (Shanghai, China). Acetone was purchased from the China National Pharmaceutical Group Corporation (Beijing, China). Methyl tertbutyl ether was purchased from CNW Technologies GmbH (Dusseldorf, Germany). NaCl and KOH were purchased from Shanghai Rhawn Chemical Technology Co., Ltd. (Shanghai, China). All of the standards (chromatographic grade, ≥95%) were purchased from Olchemim Ltd. (Olomouc, Czech Republic) and Sigma (Saint Louis, MO, USA). Formic acid was obtained from Sigma and BOC (New York, NY, USA).

### 4.2. Sample Handling

Carrots were selected based on the quality characteristics such as freshness, being pest-free, and having a uniform size. The selected tubers were surface cleaned with distilled water, peeled, and cut to 2 cm thick blocks. 

The experiment had three treatments of cut carrots: 50 mg/L ABA solution, 500 mg/L ethylene solution, and distilled water. Cut carrots were immersed with the mentioned solutions for 5 min at 25 ± 2 °C then removed from the containers and excess water was drained off and the slices were surface dried. Blocks were subsequently packed in PE film packaging, respectively. On the 0 day and 3rd day, the surface tissue of three groups of fresh cut carrot was tested for material identification and an analysis of the differences in gene expression was completed. After freezing with liquid nitrogen, the samples were stored at −80 °C.

### 4.3. Determination of Whiteness Value of Fresh Cut Carrot

The color change in the cut surface of fresh carrot was measured by chromatic aberration apparatus [30]. The whiteness index (WI) was used to represent the change in chromatic aberration, and L*, a*, and b* were measured respectively. The whiteness index is calculated by the formula: Wi = $100-\sqrt{(100-L^{*}{)}^{2}+a^{*2}+b^{*2}}$

### 4.4. Determination of Carotenoid Synthesis Pathway by Metabolome

After freeze-drying, the samples were crushed into a powder at 30 Hz for 1.5 min using a mixer mill. Then, 50 mg powdered samples were extracted by 1.0 mL mixed solution of n-hexane:acetone:ethanol (1:1:2/*v*:*v*:*v*) and internal standard [^13^C10]-β-carotene from Isoreag (Shanghai ZZBio Co., Ltd., Shanghai, China). Firstly, the samples were extracted by vortex extraction for 20 min at room temperature. The supernatant samples were evaporated to dryness under a nitrogen gas stream and reconstituted in a mixed solution of methanol:methyl tert-butyl ether (3:1/*v*:*v*), and then the solution was filtered through a 0.22 μm filter for further LC-MS analysis. The stock solutions of standards were prepared at a concentration of 1.0 mg/L. All the stock solutions were stored at −20 °C. As carotenoid quantification, different concentrations of carotenoids were prepared to construct standard curves for different carotenoids. 

The sample extracts were analyzed using an UPLC-APCI-MS /MS system (AB Sciex Ltd., Redwood City, CA, USA) [31]. The analytical conditions were as follows, LC: column, YMC C30 (3 μm, 100 mm × 2.0 mm i.d.) (YMC Co., Ltd., Tokyo, Japan); solvent system, methanol:acetonitrile (1:3, *v*/*v*) with 0.01% BHT and 0.1% formic acid (A), methyl tert-butyl ether with 0.01% BHT (B); gradient program, started at 0% B (0–3 min), increased to 70% B (3–5 min), then increased to 95% B (5–9 min), and finally ramped back to 0% B (11–12 min); flow rate of 0.8 mL min^−1^; temperature 28 °C; and injection volume: 2 uL [32].

An API 6500+Q TRAP LC/MS/MS system, equipped with an APCI Turbo lon-Spray interface, operating in a positive ion mode and controlled by an Analyst 1.6.3 software (AB Sciex), was used for the analysis. The APCI source operation parameters [33] were as follows: ion source, APCI^+^; source temperature, 350 °C; and curtain gas (CUR) was set at 25.0 psi. DP and CE for individual MRM transition was conducted with further DP and CE optimization. A transcriptional set of MRM transitions were monitored for each period according to the carotenoids eluted within this period [34].

### 4.5. Determination of Small Molecules in Lignin Pathway by Metabolome

The Freeze-dried samples were crushed using a mixer mill with a zirconia bead for 1.5 min at 30 Hz. An amount of 100 mg of powder was weighted and extracted overnight at 4 °C with 0.6 mL 70% aqueous methanol. Following centrifugation at 10,000× *g* for 10 min, the extracts were absorbed and filtrated before UPLC-MS/MS analysis.

The sample extracts were analyzed using an UPLC-ESI MS/MS system [35] (UPLC: Shimadzu, Kyoto, Japan; ESI MS/MS: Appliedbiosystems, Foster City, CA, USA). The analytical conditions were as follows, UPLC: column, Agilent SB-C18 (1.8 μm, 2.1 mm × 100 mm) (Agilent Technologies Co., Ltd., Palo Alto, CA, USA). The mobile phase consisted of 0.1% formic acid (solution A) and acetonitrile (solution B). Sample measurements were performed with a gradient program that employed the starting conditions of 95% A and 5% B. Within 9 min, a linear gradient of 5% A and 95% B was programmed and a composition of 5% A and 95% B was kept for 1 min. Subsequently, a composition of 95% A and 5% B was adjusted to 1.1 min and kept for 2.9 min. The flow rate of the moving phase was fixed at 0.35 mL/min. The column oven was set to 40 °C. The injection volume was 4 μL.

The ESI source operation parameters were as follows [36]: ion source, turbo spray; source temperature 550 °C; ion spray voltage (IS) 5500 V (positive ion mode)/−4500 V (negative ion mode); ion source gasⅠ (GSI), gasⅡ (GSⅡ), and curtain gas (CUR) were set at 50 psi, 60 psi, and 30.0 psi, respectively; and collision gas (CAD) was set at 8 psi. Instrument tuning and mass calibration were performed with 10 μmol/L and 100 μmol/L polypropylene glycol solutions in QQQ and LIT modes. QQQ scans were acquired as MRM experiments with collision gas (nitrogen) set to 5 psi.

### 4.6. Transcriptional Analysis

The qualified samples were enriched with magnetic beads with Oligo (dT) by binding the Ploy A tail of the mRNA with A-T complementary pairs. The fragmentation buffer was added to break mRNA into short pieces, using the mRNA as templates, with six bases as random primers for cDNA synthesis of a chain. Then, the buffer, dNTPs, and DNA polymerase Ⅰ cDNA synthesis chain were added by using cDNA AMPure XP beads for purification of the double chain. The purified double-stranded cDNA ends were then repaired, an A-tail was added, and sequencers were connected. Then, AMPure XP beads were used to select segment sizes. Finally, PCR enrichment was performed to obtain the final cDNA library. Agilent 2100 (Agilent Technologies Co., Ltd., Palo Alto, CA, USA) was used to detect the INSERT size of the library, and the Q-PCR method was used to accurately quantitate the effective concentration of the library. After the library was qualified, the pooling of different libraries was conducted according to the target offline data volume and the IIIumina Hiseq platform was used for sequencing [37,38,39,40]. The row RNA-seq data in this study was uploaded to the SRA database in NCBI (BioProject ID: PRJNA885975).

### 4.7. Data Processing

The technique was repeated three times for each group of samples. To determine the significance of the different treatments, analysis of variance (ANOVA) was performed using SPSS software (22.0, SPSS, Inc., Chicago, IL, USA). Treatment means were compared using Duncan’s multiple range test at the *p* ≤ 0.05 level for three independent biological experiments.

## 5. Conclusions

The whiteness of fresh-cut carrots is easily produced in natural environmental conditions and appears three days after cutting, which is a result of lignin biosynthesis. We first found that ABA and ethylene promote and inhibit the whiteness formation, respectively. The whiteness compounds were p-hydroxyphenyl lignin and guaiacyl lignin, which is involved in the phenylpropanoid biosynthesis pathway, mostly pointing to the increased expression levels of *OMT*, *AIMT*, *BMT*, *COMT*, *PAL,* and *F5H* and the decreased expression levels of *C4H*, *CCoAOMT,* and *CYP98A*. Our first findings provide new insights into the whiteness of fresh-cut carrots and lignin biosynthesis regulation, laying a research foundation for quality control of fresh-cut carrots both by chemical and transgenic methods.

## Data Availability

The data that support the findings of this study are available from the corresponding authors upon reasonable request.

## Supplementary Materials

The following supporting information can be downloaded at: https://www.mdpi.com/article/10.3390/ijms232112788/s1.
