# Supplementary material for: Characterization of Abscisic Acid and Ethylene in Regulating the White Blush in Fresh-Cut Carrots
Source: Int J Mol Sci. 2022 Oct 24;23(21):12788. doi: 10.3390/ijms232112788 (PMC9654873; doi:10.3390/ijms232112788)
Supplement: Supplementary file 1 [file ijms-23-12788-s001.zip › ijms-1916454-supplementary.pdf]

Supplementary

Table S1 Linear equation and correlation coefficient of standard curve of carotenoid substances.

Table S2 Contents of carotenoids in each group.

Table S3 Peak area of each sample substance.

Table S4 Analysis of the difference of metabolites between groups.

Figure S1 Correlation among samples

Figure S2 Volcano plot of differences in gene expression levels between groups. Down-regulated differentially expressed genes were shown in green; up-regulated differentially expressed genes were shown in red; and non-differentially expressed genes were shown in blue.

Table S1 Linear equation and correlation coefficient of standard curve of carotenoid substances

Class	Index	Equation	r	Weighting
carotenes	Phytofluene	y = 1.01893 x + 0.01762	0.99156	1 / x^2
	Phytoene	y = 0.29030 x + 0.08429	0.99321	1 / x
	ε-Carotene	y = 7.39225 x - 0.00101	0.99104	1 / x^2
	α-Carotene	y = 1.88950 x - 9.95177e^{-4}	0.99427	1 / x^2
	β-Carotene	y = 3.88371 x - 0.00451	0.99691	1 / x^2
	Lycopene	y = 0.64511 x - 6.81716e^{-4}	0.99854	1 / x
carotenoid esters	violaxanthin-laurate	y = 9.58998 x + 0.00231	0.99263	1 / x^2
	lutein-caprate	y = 9.58998 x + 0.00231	0.99263	1 / x^2
	violaxanthin-myristate	y = 9.58998 x + 0.00231	0.99263	1 / x^2
	violaxanthin-palmitate	y = 9.58998 x + 0.00231	0.99263	1 / x^2
	lutein-laurate	y = 9.58998 x + 0.00231	0.99263	1 / x^2
	lutein-myristate	y = 9.58998 x + 0.00231	0.99263	1 / x^2
	violaxanthin-dilaurate	y = 9.58998 x + 0.00231	0.99263	1 / x^2
	violaxanthin-myristate-laurate	y = 9.58998 x + 0.00231	0.99263	1 / x^2
	violaxanthin-myristate-caprate	y = 9.58998 x + 0.00231	0.99263	1 / x^2
	lutein-palmitate	y = 9.58998 x + 0.00231	0.99263	1 / x^2
	violaxanthin-dimyristate	y = 9.58998 x + 0.00231	0.99263	1 / x^2
	violaxanthin-myristate-oleate	y = 9.58998 x + 0.00231	0.99263	1 / x^2
	violaxanthin-myristate-palmitate	y = 9.58998 x + 0.00231	0.99263	1 / x^2
	lutein-dilaurate	y = 9.58998 x + 0.00231	0.99263	1 / x^2
	lutein-dioleate	y = 9.58998 x + 0.00231	0.99263	1 / x^2
	lutein-dimyristate	y = 9.58998 x + 0.00231	0.99263	1 / x^2
	violaxanthin-dipalmitate	y = 9.58998 x + 0.00231	0.99263	1 / x^2
	lutein-dipalmitate	y = 9.58998 x + 0.00231	0.99263	1 / x^2
	zeaxanthin-dipalmitate	y = 17.44435 x + 0.05158	0.99340	1 / x
	5,6epoxy-lutein-caprate-palmitate	y = 9.58998 x + 0.00231	0.99263	1 / x^2
	5,6epoxy-luttein-dilaurate	y = 9.58998 x + 0.00231	0.99263	1 / x^2
	antheraxanthin-dipalmitate	y = 9.58998 x + 0.00231	0.99263	1 / x^2
	lutein-distearate	y = 9.58998 x + 0.00231	0.99263	1 / x^2
	lutein-oleate	y = 9.58998 x + 0.00231	0.99263	1 / x^2
	lutein-stearate	y = 9.58998 x + 0.00231	0.99263	1 / x^2
	neochrome-palmitate	y = 9.58998 x + 0.00231	0.99263	1 / x^2
	rubixanthin-caprate	y = 9.58998 x + 0.00231	0.99263	1 / x^2
	rubixanthin-laurate	y = 9.58998 x + 0.00231	0.99263	1 / x^2

	rubixanthin-myristate	y = 9.58998 x + 0.00231	0.99263	1 / x^2
	rubixanthin-palmitate	y = 9.58998 x + 0.00231	0.99263	1 / x^2
	violaxanthin-dibutyrate	y = 9.58998 x + 0.00231	0.99263	1 / x^2
	violaxanthin-dioleate	y = 9.58998 x + 0.00231	0.99263	1 / x^2
	violaxanthin-palmitoleate	y = 9.58998 x + 0.00231	0.99263	1 / x^2
	zeaxanthin-caprate-laurate	y = 9.58998 x + 0.00231	0.99263	1 / x^2
	zeaxanthin-dilaurate	y = 9.58998 x + 0.00231	0.99263	1 / x^2
	zeaxanthin-dimyristate	y = 9.58998 x + 0.00231	0.99263	1 / x^2
	zeaxanthin-laurate-myristate	y = 9.58998 x + 0.00231	0.99263	1 / x^2
	zeaxanthin-laurate-palmitate	y = 9.58998 x + 0.00231	0.99263	1 / x^2
	zeaxanthin-myristate-palmitate	y = 9.58998 x + 0.00231	0.99263	1 / x^2
	zeaxanthin-myristoleate	y = 9.58998 x + 0.00231	0.99263	1 / x^2
	zeaxanthin-oleate-palmitate	y = 9.58998 x + 0.00231	0.99263	1 / x^2
	zeaxanthin-palmitate	y = 9.58998 x + 0.00231	0.99263	1 / x^2
	zeaxanthin-palmitate-stearate	y = 9.58998 x + 0.00231	0.99263	1 / x^2
	β-cryptoxanthin-laurate	y = 9.58998 x + 0.00231	0.99263	1 / x^2
	β-cryptoxanthin-myristate	y = 9.58998 x + 0.00231	0.99263	1 / x^2
	β-cryptoxanthin-oleate	y = 9.58998 x + 0.00231	0.99263	1 / x^2
	β-cryptoxanthin-palmitate	y = 9.58998 x + 0.00231	0.99263	1 / x^2
	Violaxanthin	y = 19.21851 x + 0.01079	0.99066	1 / x^2
	Neoxanthin	y = 9.58998 x + 0.00231	0.99263	1 / x^2
	β-citraurin	y = 26.97911 x + 0.00743	0.99015	1 / x^2
	Antheraxanthin	y = 3.08713 x + 0.00587	0.99127	1 / x^2
	Astaxanthin	y = 22.10576 x - 0.00623	0.9947	1 / x^2
	Lutein	y = 4.09580 x + 0.00722	0.99114	1 / x^2
	Capsorubin	y = 17.59133 x + 0.00173	0.99169	1 / x^2
xanthophylls	Capsanthin	$y = 16.56397 x + 3.28514e^{-5}$	0.99101	1 / x^2
	Apocarotenal	$y = 12.62865 x - 3.96567e^{-4}$	0.99048	1 / x^2
	Zeaxanthin	y = 1.44882 x + 0.03780	0.99237	1 / x
	canthaxanthin	y = 170.92394 x + 0.11811	0.99728	1 / x
	α-Cryptoxanthin	y = 3.43690 x - 0.00218	0.99322	1 / x^2
	echinenone	y = 104.84245 x + 0.00241	0.99077	1 / x
	β-Cryptoxanthin	y = 2.86672 x + 0.01271	0.99189	1 / x^2
	γ-Carotene	y = 2.63151 x - 0.00395	0.99632	1 / x

Table S2 Contents of carotenoids in each group

Component	Group	Mean	SD
Antheraxanthin	Eth	1.1E+00	1.08E-01
	ABA	1.1E+00	5.57E-02
	CK	1.18E+00	1.05E-01
	0 d	1.99E+00	1.04E-01
Apocarotenal	Eth	2.79E-01	2.7E-02
	ABA	2.29E-01	2.65E-02
	CK	2.28E-01	1.73E-02
	0 d	1.03E-01	1.46E-02
Lutein	Eth	1.14E+02	1.47E+01
	ABA	1.03E+02	9.41E+00
	CK	1.17E+02	7.00E+00
	0 d	1.28E+02	8.50E+00
Lycopene	Eth	1.84E+00	1.70E-01
	ABA	1.51E+00	2.26E-01
	CK	1.68E+00	1.03E-01
	0 d	1.94E+00	2.02E-01
Neoxanthin	Eth	1.26E+00	7.94E-02
	ABA	1.16E+00	1.05E-01
	CK	1.60E+00	1.00E-01
	0 d	1.65E+00	1.42E-01
Phytoene	Eth	2.79E+02	2.36E+01
	ABA	2.56E+02	2.54E+01
	CK	2.99E+02	1.49E+01
	0 d	3.79E+02	3.56E+01
Violaxanthin	Eth	7.66E-01	1.09E-01
	ABA	7.05E-01	1.01E-01
	CK	8.73E-01	6.09E-02
	0 d	1.42E+00	9.17E-02
Zeaxanthin	Eth	1.41E+00	7.37E-02
	ABA	1.07E+00	1.12E-01
	CK	8.29E-01	7.63E-02
	0 d	9.69E-01	7.66E-02
echinenone	Eth	1.65E-01	2.06E-02
	ABA	1.77E-01	9.64E-03
	CK	1.20E-01	8.39E-03
	0 d	4.35E-02	2.11E-03

lutein-caprate	Eth	6.10E-02	1.01E-02
	ABA	5.30E-02	7.19E-03
	CK	5.98E-02	1.26E-02
	0 d	4.78E-02	3.46E-03
lutein-dilaurate	Eth	2.69E+00	4.08E-01
	ABA	1.93E+00	2.97E-01
	CK	2.08E+00	3.33E-01
	0 d	2.50E+00	3.74E-01
lutein-dimyristate	Eth	7.67E+00	1.22E+00
	ABA	6.16E+00	4.27E-01
	CK	9.12E+00	1.35E+00
	0 d	9.03E+00	7.61E-01
lutein-dioleate	Eth	1.05E-01	3.13E-02
	ABA	8.81E-02	2.32E-02
	CK	1.12E-01	1.88E-02
	0 d	1.05E-01	3.16E-02
lutein-dipalmitate	Eth	7.31E-01	4.92E-02
	ABA	6.18E-01	4.86E-02
	CK	1.11E+00	1.08E-01
	0 d	8.54E-01	2.61E-02
lutein-laurate	Eth	9.33E-01	1.73E-01
	ABA	8.22E-01	8.64E-02
	CK	1.00E+00	8.45E-02
	0 d	9.03E-01	3.53E-02
lutein-myristate	Eth	4.24E+00	6.13E-01
	ABA	4.01E+00	4.97E-01
	CK	6.62E+00	4.03E-01
	0 d	5.77E+00	2.67E-01
lutein-palmitate	Eth	1.26E+00	3.06E-02
	ABA	9.80E-01	1.56E-01
	CK	2.05E+00	2.77E-01
	0 d	8.38E-01	1.07E-01
violaxanthin-dilaurate	Eth	1.49E+00	3.51E-02
	ABA	1.13E+00	1.19E-01
	CK	1.32E+00	1.99E-01
	0 d	1.54E+00	1.70E-01
violaxanthin-dimyristate	Eth	1.82E+00	2.03E-01
	ABA	1.99E+00	2.60E-01
	CK	3.48E+00	1.15E-02

	0 d	3.68E+00	3.52E-01
violaxanthin-dipalmitate	Eth	4.65E-01	5.29E-03
	ABA	4.59E-01	3.18E-02
	CK	7.24E-01	1.03E-01
	0 d	6.22E-01	3.66E-02
violaxanthin-laurate	Eth	8.83E-01	8.61E-02
	ABA	7.33E-01	6.51E-02
	CK	1.10E+00	9.85E-02
	0 d	1.03E+00	9.47E-02
violaxanthin-myristate	Eth	8.12E+00	1.34E+00
	ABA	8.44E+00	9.47E-01
	CK	1.43E+01	1.20E+00
	0 d	1.38E+01	1.08E+00
violaxanthin-myristate-caprate	Eth	1.43E+00	9.54E-02
	ABA	9.75E-01	1.34E-01
	CK	1.42E+00	7.51E-02
	0 d	1.33E+00	1.20E-01
violaxanthin-myristate-laurate	Eth	9.54E+00	1.31E+00
	ABA	7.79E+00	5.78E-01
	CK	1.12E+01	1.35E+00
	0 d	1.37E+01	1.86E+00
violaxanthin-myristate-oleate	Eth	7.82E-02	6.95E-03
	ABA	7.51E-02	1.27E-02
	CK	1.49E-01	6.56E-03
	0 d	1.31E-01	1.76E-02
violaxanthin-myristate-palmitate	Eth	1.07E+00	1.59E-01
	ABA	9.84E-01	1.35E-01
	CK	1.63E+00	1.85E-01
	0 d	1.44E+00	8.39E-02
violaxanthin-palmitate	Eth	7.75E-01	1.40E-01
	ABA	5.47E-01	9.45E-02
	CK	1.34E+00	1.44E-01
	0 d	1.11E+00	6.81E-02
α-Carotene	Eth	4.55E+02	3.64E+01
	ABA	4.56E+02	4.98E+01
	CK	3.95E+02	3.81E+01
	0 d	4.86E+02	3.80E+01
α-Cryptoxanthin	Eth	5.86E+00	7.25E-01
	ABA	5.01E+00	6.10E-01

	CK	4.73E+00	3.43E-01
	0 d	2.41E+00	2.17E-01
β-Carotene	Eth	3.67E+02	2.25E+01
	ABA	3.45E+02	3.48E+01
	CK	3.42E+02	1.95E+01
	0 d	4.31E+02	2.45E+01
β-Cryptoxanthin	Eth	1.84E+01	1.71E+00
	ABA	1.59E+01	1.85E+00
	CK	1.52E+01	7.64E-01
	0 d	4.72E+00	4.47E-01
γ-Carotene	Eth	1.94E+01	2.08E+00
	ABA	1.48E+01	2.95E+00
	CK	1.34E+01	6.56E-01
	0 d	2.23E+01	6.58E+00

Notes: ETH is the ethylene treatment group, ABA is the abscisic acid treatment group, CK is the water treatment group, all are the third day; 0 d is the surface tissue group on the 0th day of carrot extraction;

Table S3 Peak area of each sample substance

Class	Compounds	ABA_1	ABA_2	ABA_3	CK_1	CK_2	CK_3	0 d_1	0 d_2	0 d_3	Eth_1	Eth_2	Eth_3
Amino acid and derivatives	L-Phenylalanine	1.11e+07	1.08e+07	1.09e+07	8.27e+06	8.09e+06	8.01e+06	2.42e+07	2.51e+07	2.35e+07	1.10e+07	1.06e+07	1.06e+07
Phenylpropanoids	Sinapinaldehyde	4.05e+04	3.97e+04	3.91e+04	1.81e+04	1.63e+04	1.79e+04	1.23e+03	6.97e+02	1.36e+03	2.44e+04	2.35e+04	2.35e+04
	p-Coumaraldehyde	4.70e+04	4.41e+04	4.56e+04	6.65e+04	6.25e+04	5.79e+04	1.21e+03	1.48e+03	1.04e+03	9.45e+04	1.01e+05	9.44e+04
	Sinapic acid	2.32e+04	2.70e+04	2.54e+04	3.87e+04	4.42e+04	3.85e+04	3.31e+04	3.70e+04	3.29e+04	1.62e+04	1.78e+04	2.22e+04
	Coniferyl alcohol	5.42e+05	5.08e+05	5.15e+05	4.22e+05	3.99e+05	4.05e+05	1.24e+05	1.14e+05	1.26e+05	2.53e+05	2.54e+05	2.44e+05
	p-Coumaric acid	1.23e+06	1.19e+06	1.19e+06	3.49e+05	3.39e+05	3.59e+05	1.97e+05	2.24e+05	2.16e+05	4.66e+05	4.73e+05	4.58e+05
	Caffeate	1.57e+07	1.59e+07	1.55e+07	1.98e+07	1.98e+07	1.92e+07	4.26e+06	4.64e+06	4.49e+06	8.26e+06	7.44e+06	7.46e+06
	Ferulic acid	1.11e+06	1.08e+06	1.06e+06	1.28e+06	1.32e+06	1.26e+06	3.43e+04	3.74e+04	3.52e+04	2.64e+05	2.91e+05	2.75e+05
	Sinapyl alcohol	8.69e+04	7.62e+04	8.24e+04	2.96e+04	2.54e+04	2.79e+04	4.48e+04	4.85e+04	4.42e+04	3.27e+05	3.26e+05	3.15e+05
	p-Coumaryl alcohol	8.85e+05	8.21e+05	8.48e+05	6.21e+05	6.10e+05	6.18e+05	1.38e+05	1.45e+05	1.40e+05	1.15e+06	1.14e+06	1.10e+06
	4-Hydroxy-3-methoxycinnamaldehyde	2.08e+06	1.98e+06	1.99e+06	2.32e+06	2.23e+06	2.21e+06	6.97e+04	7.86e+04	8.70e+04	8.44e+05	8.35e+05	8.28e+05
	Caffeyl alcohol	N/A	N/A	N/A	N/A	N/A	N/A	N/A	N/A	N/A	N/A	N/A	N/A
	Cinnamic acid	4.32e+04	4.14e+04	4.20e+04	6.76e+04	6.58e+04	6.58e+04	3.49e+04	3.81e+04	3.60e+04	2.00e+05	1.98e+05	1.97e+05
	Caffeyl aldehyde	3.10e+05	3.14e+05	3.03e+05	2.93e+05	2.85e+05	2.88e+05	1.20e+05	1.24e+05	1.29e+05	3.18e+05	3.08e+05	3.02e+05

Table S4 Analysis of the difference of metabolites between groups

Class	Compounds	Control VS CK	Control VS ABA	Control VS Eth	ABA VS Eth	CK VS ABA	CK VS Eth
		Type	Type	Type	Type	Type	Type
Amino acid and derivatives	L-Phenylalanine	↓	↓	↓	-	-	-
Phenylpropanoids	Sinapic acid	-	-	-	-	-	↓
	Sinapyl alcohol	-	-	↑	↑	↑	↑
	Cinnamic acid	-	-	↑	↑	-	↑
	Sinapinaldehyde	↑	↑	↑	-	↑	-
	p-Coumaraldehyde	↑	↑	↑	↑	-	-
	Coniferyl alcohol	↑	↑	↑	↓	-	-
	p-Coumaric acid	-	↑	↑	↓	↑	-
	Caffeate	↑	↑	-	↓	-	↓
	Ferulic acid	↑	↑	↑	↓	-	↓
	p-Coumaryl alcohol	↑	↑	↑	-	-	-
	4-Hydroxy-3-methoxycinnamaldehyde	↑	↑	↑	↓	-	↓
	Caffeyl aldehyde	↑	↑	↑	-	-	-

↓ : the metabolite content was significantly down; -: the metabolite content had no significant change; ↑ : the metabolite content was significantly up.

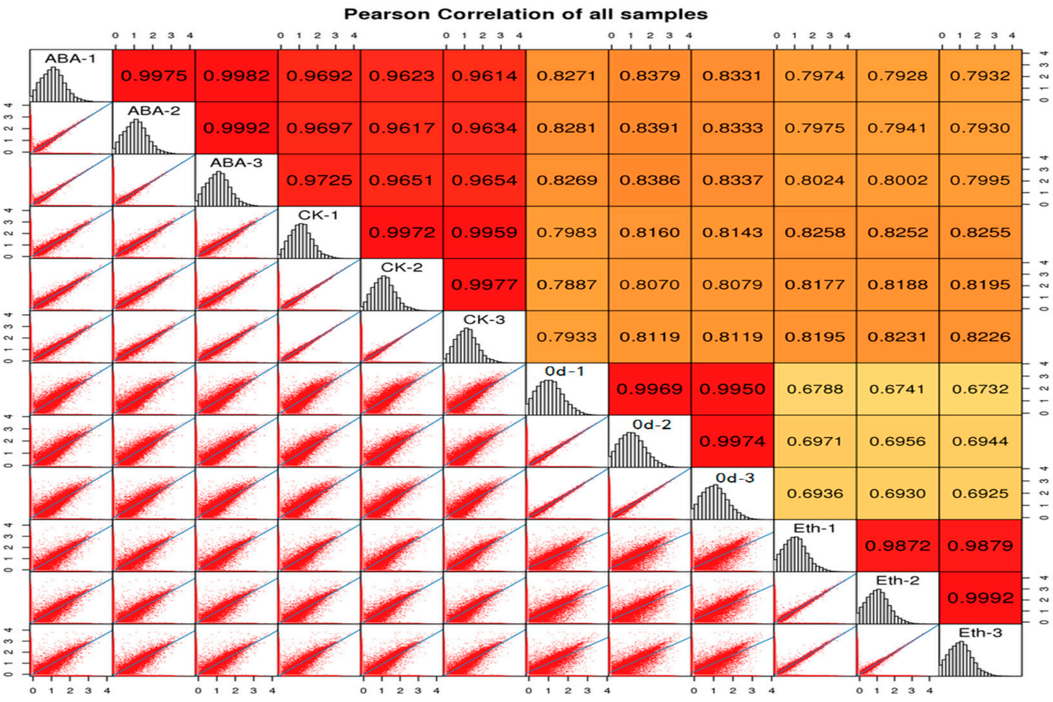

Fig. S1 Correlation among samples

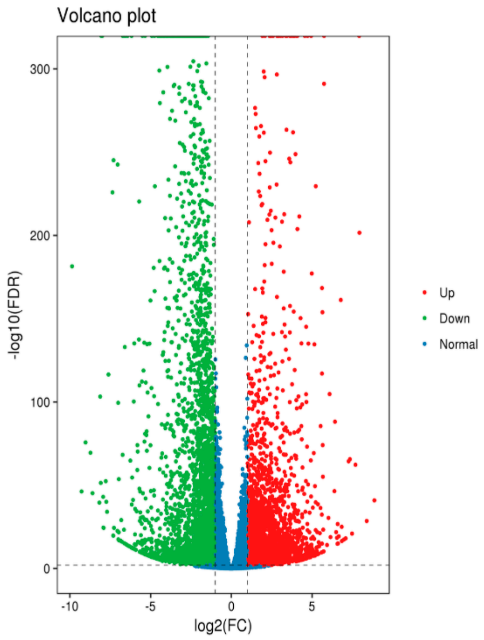

A- the difference between 0 day and CK gene expression levels

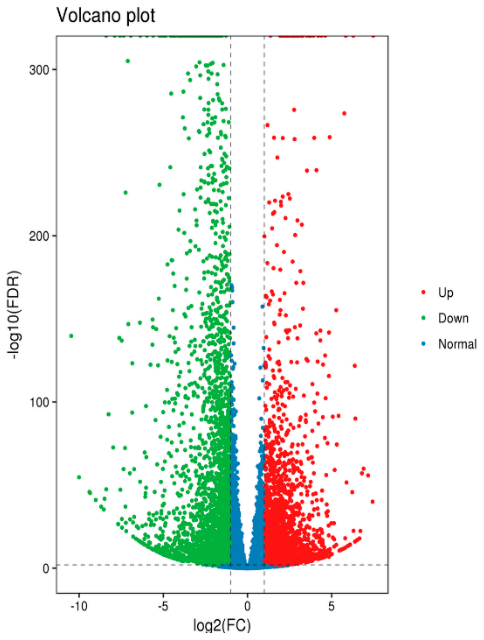

B- the difference between 0 day and ABA gene expression levels

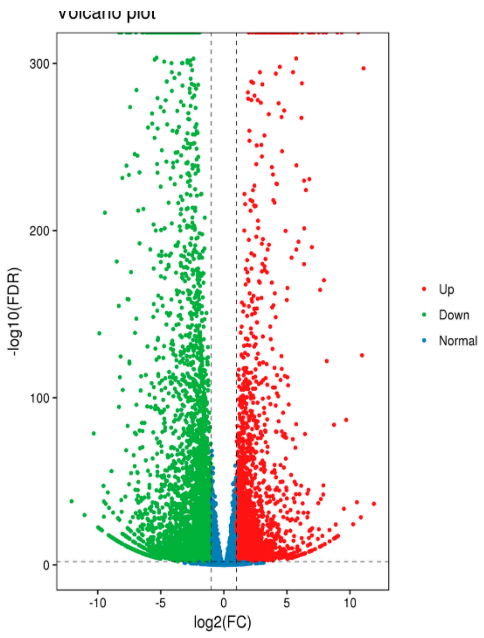

C- the difference between 0 day and Eth gene expression levels

Fig. S2 Volcano plot of differences in gene expression levels between groups

Down-regulated differentially expressed genes were shown in green; up-regulated differentially expressed genes were shown in red; and non-differentially expressed genes were shown in blue.
